# (2-Chloro-3,5-dinitro­phen­yl)(piperidin-1-yl)methanone

**DOI:** 10.1107/S1600536811011469

**Published:** 2011-04-07

**Authors:** Xun Luo, Yun-Chuang Huang, Chao Gao, Luo-Ting Yu

**Affiliations:** a State Key Laboratory of Biotherapy and Cancer Center, West China Hospital, West China Medical School, Sichuan University, Chengdu 610041, People’s Republic of China

## Abstract

In the title compound, C_12_H_12_ClN_3_O_5_, the piperidine ring adopts a chair conformation. One of the nitro groups is almost coplanar with the aromatic ring [O—N—C—C = −1.4 (2)°], whereas the other one is significantly twisted out of the ring plane [O—N—C—C = 34.7 (2)°]. The crystal packing is stabilized by inter­molecular π–π stacking inter­actions with centroid–centroid distances of 3.579 (3) Å.

## Related literature

For the biological activity of benzamide derivatives, see: Christophe *et al.* (2009 ▶).
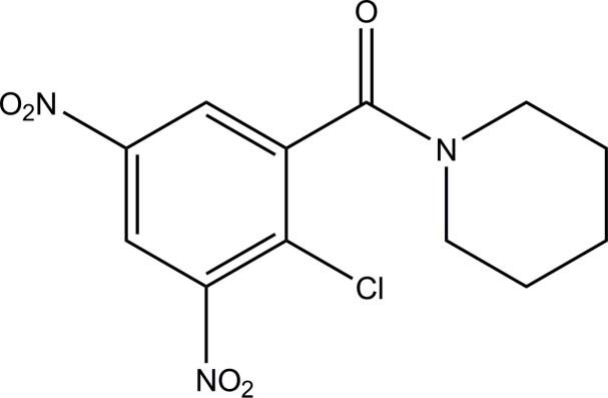


## Experimental

### 

#### Crystal data

C_12_H_12_ClN_3_O_5_
*M* *_r_* = 313.70
Orthorhombic, 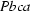

*a* = 10.7864 (3) Å
*b* = 11.1264 (3) Å
*c* = 21.9775 (5) Å
*V* = 2637.60 (11) Å^3^
*Z* = 8
Mo *K*α radiation
μ = 0.32 mm^−1^
*T* = 150 K
0.40 × 0.38 × 0.20 mm

#### Data collection

Oxford Diffraction Xcalibur Eos diffractometer
Absorption correction: multi-scan (*CrysAlis PRO*; Oxford Diffraction, 2006 ▶) *T*_min_ = 0.979, *T*_max_ = 1.0
6722 measured reflections
2696 independent reflections
2240 reflections with *I* > 2σ(*I*)
*R*_int_ = 0.021

#### Refinement

*R*[*F* ^2^ > 2σ(*F* ^2^)] = 0.033
*wR*(*F* ^2^) = 0.077
*S* = 1.05
2696 reflections
238 parameters
All H-atom parameters refined
Δρ_max_ = 0.25 e Å^−3^
Δρ_min_ = −0.24 e Å^−3^

### 

Data collection: *CrysAlis PRO* (Oxford Diffraction, 2006 ▶); cell refinement: *CrysAlis PRO*; data reduction: *CrysAlis PRO*; program(s) used to solve structure: *SHELXS97* (Sheldrick, 2008 ▶); program(s) used to refine structure: *SHELXL97* (Sheldrick, 2008 ▶); molecular graphics: *OLEX2* (Dolomanov *et al.*, 2009 ▶); software used to prepare material for publication: *OLEX2*.

## Supplementary Material

Crystal structure: contains datablocks I, global. DOI: 10.1107/S1600536811011469/bt5492sup1.cif

Structure factors: contains datablocks I. DOI: 10.1107/S1600536811011469/bt5492Isup2.hkl

Additional supplementary materials:  crystallographic information; 3D view; checkCIF report

## Figures and Tables

**Fig. 1.**
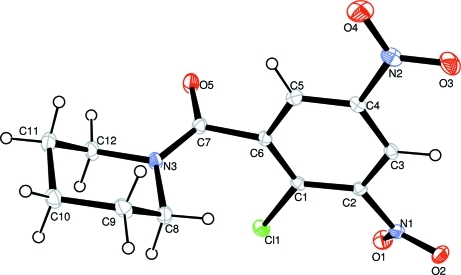
The molecular structure of the title compound, with displacement ellipsoids drawn at the 50% probability level.

**Fig. 2.**
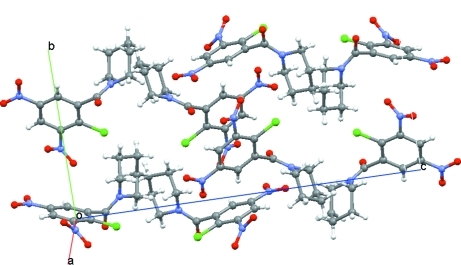
A packing diagram of the title compound.

## supplementary crystallographic information

### Comment

Benzamide derivatives are of great importance owing to their antibacterial properties (Christophe *et al.*, 2009). The title compound is one of the key intermediates in our synthetic investigations of antibacterial drugs.

The piperidine ring adopts a chair conformation. As shown in Fig.1, the amide group forms a dihedral angles of 75.96 (5)° and 51.61 (9)° with the benzene ring and the piperidine ring, respectively. The crystal packing is strengthened by intermolecular π–π stacking interaction with centroid–centroid distances of 3.579 (3) Å.

### Experimental

A solution of 3.42 g (12.9 mmol) of 2-chloro-3,5-dinitrobenzoyl chloride in 20 ml of dichloromethane was added to a solution of 1.097 g (12.9 mmol) piperidine with a catalyst of 1.82 g (17.9 mmol) triethylamine. The mixture was stirred for 2 h at room temperature and extracted with water and dichloromethane, then the organic solvent was evaporated and the title compound was recrystallized from ethanol (yield 3.24 g, 80%). Crystals suitable for X-ray analysis were obtained by slow evaporation from a solution of ethyl acetate.

### Crystal data

**Table:**

C_12_H_12_ClN_3_O_5_	*F*(000) = 1296
*M**_r_* = 313.70	*D*_x_ = 1.580 Mg m^−^^3^
Orthorhombic, *P**b**c**a*	Mo *K*α radiation, λ = 0.7107 Å
Hall symbol: -P 2ac 2ab	Cell parameters from 3316 reflections
*a* = 10.7864 (3) Å	θ = 3.2–29.1°
*b* = 11.1264 (3) Å	µ = 0.32 mm^−^^1^
*c* = 21.9775 (5) Å	*T* = 150 K
*V* = 2637.60 (11) Å^3^	Block, yellow
*Z* = 8	0.40 × 0.38 × 0.20 mm

### Data collection

**Table:**

Oxford Diffraction Xcalibur Eos diffractometer	2696 independent reflections
Radiation source: fine-focus sealed tube	2240 reflections with *I* > 2σ(*I*)
graphite	*R*_int_ = 0.021
Detector resolution: 16.0874 pixels mm^-1^	θ_max_ = 26.4°, θ_min_ = 3.2°
ω scans	*h* = −13→7
Absorption correction: multi-scan (*CrysAlis PRO*; Oxford Diffraction, 2006)	*k* = −13→13
*T*_min_ = 0.979, *T*_max_ = 1.0	*l* = −27→15
6722 measured reflections	

### Refinement

**Table:**

Refinement on *F*^2^	Primary atom site location: structure-invariant direct methods
Least-squares matrix: full	Secondary atom site location: difference Fourier map
*R*[*F*^2^ > 2σ(*F*^2^)] = 0.033	Hydrogen site location: inferred from neighbouring sites
*wR*(*F*^2^) = 0.077	All H-atom parameters refined
*S* = 1.05	*w* = 1/[σ^2^(*F*_o_^2^) + (0.0283*P*)^2^ + 0.9862*P*] where *P* = (*F*_o_^2^ + 2*F*_c_^2^)/3
2696 reflections	(Δ/σ)_max_ < 0.001
238 parameters	Δρ_max_ = 0.25 e Å^−^^3^
0 restraints	Δρ_min_ = −0.24 e Å^−^^3^

### Special details

**Table:**

Geometry. All e.s.d.'s (except the e.s.d. in the dihedral angle between two l.s. planes) are estimated using the full covariance matrix. The cell e.s.d.'s are taken into account individually in the estimation of e.s.d.'s in distances, angles and torsion angles; correlations between e.s.d.'s in cell parameters are only used when they are defined by crystal symmetry. An approximate (isotropic) treatment of cell e.s.d.'s is used for estimating e.s.d.'s involving l.s. planes.
Refinement. Refinement of *F*^2^ against ALL reflections. The weighted *R*-factor *wR* and goodness of fit *S* are based on *F*^2^, conventional *R*-factors *R* are based on *F*, with *F* set to zero for negative *F*^2^. The threshold expression of *F*^2^ > σ(*F*^2^) is used only for calculating *R*-factors(gt) *etc*. and is not relevant to the choice of reflections for refinement. *R*-factors based on *F*^2^ are statistically about twice as large as those based on *F*, and *R*- factors based on ALL data will be even larger.

### Fractional atomic coordinates and isotropic or equivalent isotropic displacement parameters (Å^2^)

**Table:**

	*x*	*y*	*z*	*U*_iso_*/*U*_eq_	
Cl1	0.14710 (4)	0.51437 (4)	0.126198 (18)	0.02618 (12)	
O1	0.28382 (11)	0.39665 (10)	0.03083 (6)	0.0308 (3)	
O2	0.37259 (10)	0.52238 (11)	−0.03080 (6)	0.0297 (3)	
O3	0.06436 (13)	0.74355 (12)	−0.14870 (6)	0.0376 (3)	
O4	−0.09872 (13)	0.81129 (12)	−0.10265 (6)	0.0409 (4)	
O5	−0.18116 (11)	0.60708 (11)	0.10878 (6)	0.0331 (3)	
N1	0.28706 (12)	0.49108 (12)	0.00224 (6)	0.0219 (3)	
N2	−0.00012 (14)	0.75653 (13)	−0.10356 (7)	0.0269 (3)	
N3	−0.05433 (13)	0.73621 (12)	0.15851 (6)	0.0238 (3)	
C1	0.10865 (14)	0.58412 (14)	0.05898 (7)	0.0179 (3)	
C2	0.17880 (14)	0.57192 (13)	0.00600 (7)	0.0178 (3)	
C3	0.14762 (15)	0.63140 (14)	−0.04708 (7)	0.0188 (3)	
H3	0.1955 (17)	0.6182 (15)	−0.0830 (8)	0.028 (5)*	
C4	0.04113 (15)	0.69969 (13)	−0.04645 (7)	0.0191 (3)	
C5	−0.03125 (16)	0.71333 (14)	0.00470 (7)	0.0204 (4)	
H5	−0.1055 (16)	0.7566 (15)	0.0036 (7)	0.018 (4)*	
C6	0.00352 (14)	0.65690 (13)	0.05846 (7)	0.0177 (3)	
C7	−0.08451 (15)	0.66492 (14)	0.11200 (7)	0.0207 (3)	
C8	0.05941 (16)	0.80784 (16)	0.16249 (9)	0.0274 (4)	
H8A	0.1068 (18)	0.7963 (17)	0.1263 (9)	0.034 (5)*	
H8B	0.1111 (17)	0.7754 (16)	0.1984 (9)	0.032 (5)*	
C9	0.02738 (18)	0.93938 (16)	0.17196 (8)	0.0274 (4)	
H9A	−0.0143 (18)	0.9693 (16)	0.1353 (9)	0.031 (5)*	
H9B	0.1038 (18)	0.9841 (16)	0.1769 (8)	0.029 (5)*	
C10	−0.05450 (17)	0.95620 (17)	0.22745 (8)	0.0274 (4)	
H10A	−0.0069 (16)	0.9325 (15)	0.2639 (8)	0.025 (5)*	
H10B	−0.0785 (17)	1.0388 (17)	0.2320 (8)	0.033 (5)*	
C11	−0.16990 (17)	0.87786 (17)	0.22253 (9)	0.0306 (4)	
H11A	−0.2230 (19)	0.9067 (18)	0.1888 (9)	0.040 (6)*	
H11B	−0.2193 (18)	0.8836 (16)	0.2593 (10)	0.041 (6)*	
C12	−0.13634 (17)	0.74677 (16)	0.21155 (8)	0.0269 (4)	
H12A	−0.2103 (16)	0.7001 (15)	0.2041 (8)	0.022 (4)*	
H12B	−0.0908 (17)	0.7123 (15)	0.2473 (9)	0.028 (5)*	

### Atomic displacement parameters (Å^2^)

**Table:**

	*U*^11^	*U*^22^	*U*^33^	*U*^12^	*U*^13^	*U*^23^
Cl1	0.0277 (2)	0.0334 (2)	0.0174 (2)	0.00122 (18)	−0.00259 (17)	0.00550 (16)
O1	0.0322 (7)	0.0251 (6)	0.0350 (7)	0.0091 (6)	−0.0015 (6)	0.0029 (6)
O2	0.0189 (6)	0.0370 (7)	0.0332 (7)	−0.0013 (5)	0.0053 (5)	−0.0070 (6)
O3	0.0446 (8)	0.0466 (8)	0.0216 (7)	−0.0021 (7)	−0.0007 (6)	0.0093 (6)
O4	0.0399 (8)	0.0395 (8)	0.0434 (8)	0.0123 (7)	−0.0068 (7)	0.0168 (7)
O5	0.0250 (6)	0.0402 (7)	0.0341 (7)	−0.0139 (6)	0.0082 (6)	−0.0182 (6)
N1	0.0188 (7)	0.0246 (7)	0.0222 (7)	0.0011 (6)	−0.0029 (6)	−0.0045 (6)
N2	0.0329 (8)	0.0232 (7)	0.0246 (8)	−0.0050 (7)	−0.0055 (7)	0.0064 (6)
N3	0.0220 (7)	0.0285 (7)	0.0209 (7)	−0.0096 (6)	0.0061 (6)	−0.0079 (6)
C1	0.0199 (7)	0.0175 (8)	0.0162 (7)	−0.0048 (7)	−0.0022 (7)	0.0008 (6)
C2	0.0172 (7)	0.0166 (8)	0.0198 (8)	−0.0012 (7)	−0.0015 (6)	−0.0035 (6)
C3	0.0215 (8)	0.0191 (8)	0.0159 (8)	−0.0049 (7)	0.0011 (7)	−0.0026 (6)
C4	0.0244 (8)	0.0141 (7)	0.0187 (8)	−0.0030 (7)	−0.0036 (7)	0.0014 (6)
C5	0.0203 (8)	0.0154 (8)	0.0256 (9)	0.0010 (7)	−0.0010 (7)	−0.0022 (7)
C6	0.0193 (7)	0.0151 (7)	0.0186 (8)	−0.0038 (6)	0.0001 (7)	−0.0048 (6)
C7	0.0205 (8)	0.0189 (8)	0.0228 (9)	−0.0007 (7)	0.0018 (7)	−0.0032 (7)
C8	0.0225 (9)	0.0332 (10)	0.0266 (10)	−0.0106 (8)	0.0062 (8)	−0.0110 (8)
C9	0.0303 (10)	0.0295 (10)	0.0224 (9)	−0.0119 (8)	0.0016 (8)	−0.0023 (7)
C10	0.0308 (9)	0.0256 (9)	0.0258 (9)	−0.0027 (8)	0.0018 (8)	−0.0077 (8)
C11	0.0261 (9)	0.0381 (11)	0.0277 (10)	−0.0018 (8)	0.0076 (8)	−0.0098 (8)
C12	0.0272 (9)	0.0322 (9)	0.0212 (9)	−0.0100 (8)	0.0084 (8)	−0.0058 (8)

### Geometric parameters (Å, °)

**Table:**

Cl1—C1	1.7196 (16)	C5—H5	0.935 (17)
O1—N1	1.2247 (17)	C5—C6	1.389 (2)
O2—N1	1.2246 (17)	C6—C7	1.515 (2)
O3—N2	1.2200 (19)	C8—H8A	0.95 (2)
O4—N2	1.2260 (19)	C8—H8B	1.032 (19)
O5—C7	1.2273 (19)	C8—C9	1.518 (3)
N1—C2	1.476 (2)	C9—H9A	0.98 (2)
N2—C4	1.474 (2)	C9—H9B	0.97 (2)
N3—C7	1.334 (2)	C9—C10	1.517 (2)
N3—C8	1.466 (2)	C10—H10A	0.987 (18)
N3—C12	1.468 (2)	C10—H10B	0.960 (19)
C1—C2	1.395 (2)	C10—C11	1.523 (2)
C1—C6	1.394 (2)	C11—H11A	0.99 (2)
C2—C3	1.383 (2)	C11—H11B	0.97 (2)
C3—H3	0.954 (18)	C11—C12	1.522 (3)
C3—C4	1.377 (2)	C12—H12A	0.966 (17)
C4—C5	1.377 (2)	C12—H12B	1.004 (19)
			
O1—N1—C2	118.12 (13)	C6—C1—Cl1	117.75 (12)
O2—N1—O1	124.73 (14)	C6—C1—C2	119.41 (14)
O2—N1—C2	117.11 (13)	C6—C5—H5	119.0 (10)
O3—N2—O4	124.53 (15)	C7—N3—C8	124.97 (14)
O3—N2—C4	118.00 (14)	C7—N3—C12	120.59 (13)
O4—N2—C4	117.45 (15)	C8—N3—C12	114.43 (13)
O5—C7—N3	124.28 (15)	C8—C9—H9A	108.6 (11)
O5—C7—C6	117.18 (14)	C8—C9—H9B	108.5 (10)
N3—C7—C6	118.51 (13)	H8A—C8—H8B	107.6 (15)
N3—C8—H8A	109.0 (11)	C9—C8—H8A	111.5 (12)
N3—C8—H8B	107.9 (10)	C9—C8—H8B	110.8 (10)
N3—C8—C9	109.99 (15)	C9—C10—H10A	108.5 (10)
N3—C12—C11	110.24 (15)	C9—C10—H10B	111.1 (11)
N3—C12—H12A	108.7 (10)	C9—C10—C11	110.37 (15)
N3—C12—H12B	107.2 (10)	H9A—C9—H9B	107.9 (15)
C1—C2—N1	122.34 (14)	C10—C9—C8	111.20 (15)
C1—C6—C7	122.55 (14)	C10—C9—H9A	110.6 (11)
C2—C1—Cl1	122.84 (12)	C10—C9—H9B	110.0 (11)
C2—C3—H3	119.5 (11)	C10—C11—H11A	109.9 (12)
C3—C2—N1	115.90 (14)	C10—C11—H11B	110.6 (11)
C3—C2—C1	121.70 (14)	H10A—C10—H10B	108.1 (15)
C3—C4—N2	118.68 (14)	C11—C10—H10A	109.2 (10)
C4—C3—C2	117.30 (15)	C11—C10—H10B	109.5 (11)
C4—C3—H3	122.9 (11)	C11—C12—H12A	110.2 (10)
C4—C5—H5	121.5 (10)	C11—C12—H12B	111.0 (10)
C4—C5—C6	119.43 (15)	H11A—C11—H11B	106.6 (16)
C5—C4—N2	118.47 (14)	C12—C11—C10	111.42 (15)
C5—C4—C3	122.80 (15)	C12—C11—H11A	109.2 (12)
C5—C6—C1	119.29 (14)	C12—C11—H11B	109.0 (11)
C5—C6—C7	117.69 (14)	H12A—C12—H12B	109.3 (14)
			
Cl1—C1—C2—N1	4.4 (2)	C2—C3—C4—N2	−175.24 (14)
Cl1—C1—C2—C3	−178.34 (12)	C2—C3—C4—C5	2.1 (2)
Cl1—C1—C6—C5	−179.12 (12)	C3—C4—C5—C6	0.3 (2)
Cl1—C1—C6—C7	−7.17 (19)	C4—C5—C6—C1	−2.2 (2)
O1—N1—C2—C1	34.7 (2)	C4—C5—C6—C7	−174.59 (14)
O1—N1—C2—C3	−142.65 (15)	C5—C6—C7—O5	71.21 (19)
O2—N1—C2—C1	−147.16 (15)	C5—C6—C7—N3	−106.95 (18)
O2—N1—C2—C3	35.45 (19)	C6—C1—C2—N1	−176.46 (13)
O3—N2—C4—C3	−2.2 (2)	C6—C1—C2—C3	0.8 (2)
O3—N2—C4—C5	−179.72 (14)	C7—N3—C8—C9	123.65 (18)
O4—N2—C4—C3	176.04 (14)	C7—N3—C12—C11	−124.47 (17)
O4—N2—C4—C5	−1.4 (2)	C8—N3—C7—O5	−178.63 (17)
N1—C2—C3—C4	174.76 (13)	C8—N3—C7—C6	−0.6 (2)
N2—C4—C5—C6	177.67 (14)	C8—N3—C12—C11	56.3 (2)
N3—C8—C9—C10	55.5 (2)	C8—C9—C10—C11	−55.0 (2)
C1—C2—C3—C4	−2.6 (2)	C9—C10—C11—C12	54.1 (2)
C1—C6—C7—O5	−100.86 (19)	C10—C11—C12—N3	−53.7 (2)
C1—C6—C7—N3	81.0 (2)	C12—N3—C7—O5	2.2 (3)
C2—C1—C6—C5	1.7 (2)	C12—N3—C7—C6	−179.80 (15)
C2—C1—C6—C7	173.67 (14)	C12—N3—C8—C9	−57.1 (2)
